# Une énorme mucocèle orbitaire

**DOI:** 10.11604/pamj.2014.18.228.4778

**Published:** 2014-07-18

**Authors:** Fatima Zohra El Meriague, Rajae Daoudi

**Affiliations:** 1 Université Mohamed V, Souissi, Service d'Ophtalmologie A de l'hôpital des Spécialités, Centre Hospitalier Universitaire, Rabat, Maroc

**Keywords:** Mucocèle, cavité orbitaire, globe oculaire, Mucocele, eye socket, eye-ball

## Image en medicine

La mucocèle orbitaire est une tumeur sinusienne bénigne qui se manifeste par une masse palpable refoulant le globe oculaire. Cette photo illustre l'importance que peut prendre cette masse. Le diagnostic doit être fait de manière précoce et le traitement adapté pour ne pas arriver à ce stade. Il s'agit d'un patient de 50 ans, sans antécédents pathologiques particuliers, qui présente une énorme mucocèle orbitaire à point de départ ethmoïdal. L'examen ophtalmologique montre une exophtalmie non inflammatoire non axile et une énorme masse orbitaire refoulant le globe oculaire en bas et en dehors. L'acuité visuelle était à 10/10, le TO à 22 mmhg, la cornée est claire, la CA est optiquement vide. Le fond d'oeil montre des plis rétiniens. L'IRM orbitocérébrale montre une énorme masse orbitaire hyperdense à point de départ ethmoïdal. La biopsie a confirmé le diagnostic de mucocèle. Le traitement a consisté en l’évacuation de son contenu, la résection de la coque et d'une ventilation normale du sinus par drainage par voie endonasale.

**Figure 1:**
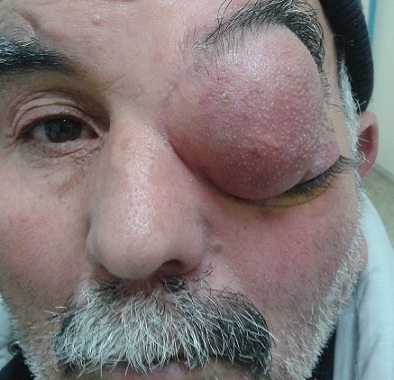
Énorme mucocèle orbitaire refoulant le globe oculaire en bas et en dehors

